# Protecting health workers from infectious disease transmission: an exploration of a Canadian-South African partnership of partnerships

**DOI:** 10.1186/s12992-016-0145-0

**Published:** 2016-03-31

**Authors:** Annalee Yassi, Muzimkhulu Zungu, Jerry M. Spiegel, Barry Kistnasamy, Karen Lockhart, David Jones, Lyndsay M. O’Hara, Letshego Nophale, Elizabeth A. Bryce, Lincoln Darwin

**Affiliations:** Global Health Research Program (GHRP), The University of British Columbia (UBC), Rm. 430, 2206 East Mall, V6T 1Z3 Vancouver, BC Canada; National Institute for Occupational Health (NIOH), a division of National Health Laboratory Service (NHLS), Johannesburg, South Africa; University of Pretoria, School of Health Systems and Public Health, Pretoria, South Africa; Department of Health, Compensation Commissioner, Johannesburg, South Africa; Provincial Occupational Health Unit and Centre for Health Systems Research & Development, University of the Free State (UFS), Bloemfontein, South Africa; Vancouver General Hospital (VGH), Vancouver Coastal Health, Vancouver, BC Canada

**Keywords:** Partnership, Community of practice, North–South, North–South-South, Health worker, Occupational health, Infection control, South Africa, Tuberculosis

## Abstract

**Background:**

Health workers are at high risk of acquiring infectious diseases at work, especially in low and middle-income countries (LMIC) with critical health human resource deficiencies and limited implementation of occupational health and infection control measures. Amidst increasing interest in international partnerships to address such issues, how best to develop such collaborations is being actively debated. In 2006, a partnership developed between occupational health and infection control experts in Canada and institutions in South Africa (including an institute with a national mandate to conduct research and provide guidance to protect health workers from infectious diseases and promote improved working conditions). This article describes the collaboration, analyzes the determinants of success and shares lessons learned.

**Methods:**

Synthesizing participant-observer experience from over 9 years of collaboration and 10 studies already published from this work, we applied a realist review analysis to describe the various achievements at global, national, provincial and hospital levels. Expectations of the various parties on developing new insights, providing training, and addressing service needs were examined through a micro-meso-macro lens, focusing on how each main partner organization contributed to and benefitted from working together.

**Results:**

A state-of-the-art occupational health and safety surveillance program was established in South Africa following successful technology transfer from a similar undertaking in Canada and training was conducted that synergistically benefitted Northern as well as Southern trainees. Integrated policies combining infection control and occupational health to prevent and control infectious disease transmission among health workers were also launched. Having a national (South-South) network reinforced by the international (North–south) partnership was pivotal in mitigating the challenges that emerged.

**Conclusions:**

High-income country partnerships with experience in health system strengthening – particularly in much needed areas such as occupational health and infection control – can effectively work through strong collaborators in the Global South to build capacity. Partnerships are particularly well positioned to sustainably reinforce efforts at national and sub-national LMIC levels when they adopt a “communities of practice” model, characterized by multi-directional learning. The principles of effective collaboration learned in this “partnership of partnerships” to improve working conditions for health workers can be applied to other areas where health system strengthening is needed.

## Background

The 2013 report of the Third Global Forum on Human Resources for Health (HRH) observed that “in many countries, the employment conditions of health workers are not compatible with the attainment of universal health coverage.” It specifically noted that “in some settings, working conditions are characterized by understaffing, excessive workloads, stress, exposure to occupational hazards, unsafe environments, occupational ill health and violence, resulting in inadequate patient care”[1]. In low- and middle-income countries (LMICs) where shortfalls in HRH are especially severe [2, 3], such circumstances contribute to migration [4, 5] and departure from public sector facilities [6, 7]. Just as peer-to-peer North–south partnerships between health practitioners promote *clinical *skill development of health workers in LMICs [8, 9], similar collaborations are also desperately needed to build capacities for improving LMIC *work environments* in healthcare. How to develop, conduct and sustain the benefits of such international partnerships is the subject of active debate [10–14].

The protection of health workers’ health has tended to be neglected globally, however in high-income countries (HICs) the much lower prevalence of transmissible communicable diseases and better infrastructure has mitigated the impact of occupational exposure. For example, the personal protective equipment and needed training to prevent occupational respiratory infections is often lacking in LMICs [15–17], but generally available in HICs [18, 19]. While the more favourable access to resources has created opportunities for technical and organizational innovation, including the development of information systems [20], it is important to recognize that expertise and access to technological innovation is rapidly growing within emerging economies as well [13]. Noting the prospects for adapting such experiences for settings of high need, in 2006 two World Health Organization (WHO) Collaborating Centres for Occupational Health (one in South Africa and one in Canada) initiated collaboration with this objective.

Health workers are especially at risk of exposure to infectious diseases. Canada took sharp note of this in the SARS outbreak of 2003 when almost half the cases occurred in health workers [21]. It has also been estimated that 40 % of the Hepatitis B and C cases in health workers are likely due to occupational exposures [22–24]. Health workers have a high risk of tuberculosis (TB) [25], and, most recently, a high rate of Ebola was documented in health workers [26]. The elevated risk of TB in health workers [15, 27, 28] was highlighted by recent TB outbreaks in South African hospitals [28–30], with some studies suggesting that health workers are three to ten times more likely to acquire TB [31]. For multiple drug resistant TB (MDR-TB), the risk is even higher- with an estimated incidence of 64.8 per 100,000 health workers compared with 11.9 per 100,000 general population in South Africa between 2003 and 2008 [25]. Similarly, the estimated incidence of extreme drug resistant TB (XDR-TB) was 7.2 per 100,000 health workers compared with 1.1 per 100,000 general population between 2003 and 2008 [25]. Furthermore, there is considerable evidence that prevention and control of infectious disease among health workers is not only a benefit in itself, but is a significant contributor to patient safety [32].

Exposures are generally preventable with prompt identification and isolation of potentially infectious patients; selection and use of appropriate personal protective equipment (PPE); immediate and safe procedures for cleaning up blood and body fluid spills; correctly disposing of contaminated sharps and biomedical waste; adherence to routine immunisations; and consistent practice of respiratory etiquette and hand hygiene. Advisories, notably *the Joint WHO-ILO-UNAIDS Policy Guidelines on Improving Health Care Workers’ Access to HIV and TB Prevention, Treatment, Care and Support* [33] emphasise the importance of strengthening infection control programmes and ensuring a safe working environment for health workers. The importance of occupational health-infection control collaboration was highlighted in these [33] and in general infection control guidelines [34].

Brinkerhoff observed that while international partnerships can provide a ‘rational response to complexity’ that can build on comparative advantages and divisions of labour [35], they unavoidably also mirror “dimensions of power, participation, trust and sustainability, as well as mutuality” – the latter also emphasized by Johnson and Wilson [10]. According to Corbin and colleagues [36], North–South partnerships have replaced older models of aid and development by giving hope that such a partnership would link Northern money and expertise with Southern know-how and community participation to create relevant local health and development initiatives. The opportunities to broaden the notion of “capacity-building” in response to complex challenges are still in early phases of critical assessment. The challenge to more comprehensively embrace the concepts of joint learning and knowledge transfer has encouraged consideration of “community of practice” approaches that actively encompass different types of knowledge and experience [11]. This approach attempts to reduce the power imbalances discussed by Holmarsdottir, Desai, Botha, Breidlid and colleagues [12].

To contribute to this debate, the research questions we address in this article are, first, what partnership model characterizes our collaboration linking Canadian and South African infection control and occupational health professionals? Secondly, what contributed to the successes we achieved? And thirdly, what lessons can be drawn about partnership models?

Our partnership aimed to build capacity in South Africa as well as in Canada to address the linked area of occupational health and infection control. Indeed we trained dozens of health workers in South Africa; produced guidelines, policies and procedures; and co-developed a health information system (based on one developed in Canada [20, 37]) that has been implemented across South Africa as part of a process of building capacity of healthcare workers and administrators in that country. Notably, re-enforcing the conclusions of Johnson and Wilson [10] for example related to the mutual benefit of such endeavours, and the importance of learning from the Global South (for example, Spiegel et al.,[38]), we also built capacity of over a dozen Northern research trainees and acquired considerable insights of benefit to the Northern as well as Southern partners. The usefulness to Canada of this international collaboration was recognized by an award given by Canada’s top medical authorities to two of the Canadian practitioners involved [39, 40]. Our findings also support the call (for example, Holmarsdottir et al, [12]) to challenge hegemonic knowledge-production that has characterized many previous North–South collaborations; our experience indeed stresses the importance of respecting Southern perspectives and Southern leadership within a North–South-South community of practice.

## Methods

### Conceptual approach

In order to address the first question and discern how to characterize our partnership, we begin by introducing the collaboration in Canada between university-based and hospital-based personnel, analyzing its key features with respect to contributing to a global partnership. Next, we describe the context in which this Canadian group developed partnerships in South Africa, and, finally we characterize the approach adopted at the national, provincial, and hospital levels. To address the second research question, we present specific activities undertaken by the partnership, highlighting challenges as well as outcomes, paying special attention to how the varied expectations of the different parties within the collaboration were met. To analyze “what contributes to success” in our partnership, we used a realist review perspective, namely analyzing the context, mechanism and outcome of each endeavour we undertook, identifying the micro, meso and macro scale processes involved [26, 38]. Specifically our approach examined: i) the micro context – ascertaining what mechanisms determined *individuals’* preparedness to address potential health and safety risks, as well as their readiness to participate in processes designed to ensure their right to a secure environment; ii) the meso context – ascertaining the mechanism by which *workplace* managers were provided with infrastructural support to meet this challenge in collaboration with worker representatives; and iii) the macro context – especially ascertaining mechanisms used and outcomes achieved at the level of the *provincial and national health departments*. We present specific activities undertaken by the partnership, highlighting challenges as well as outcomes, applying our micro-meso-macro framework to analyze the mechanisms that led to these, paying special attention to how the varied expectations of the different parties within the collaboration were met.

Each of the specific studies conducted by the partnership had its own ethics-approved protocol, with detailed sections on the methodologies employed. Ethics approval for all associated research activity was obtained from the UBC Behavioural Research Ethics Board, University of Free State Ethics Board, Research Ethics Committee, Faculty of Health Sciences, University of Pretoria, in addition to the approval of the National Department of Health (DoH) Free State DoH, Gauteng DoH and the various hospitals involved. As this article constitutes a meta-analysis of the studies conducted, a separate ethics approval was not deemed necessary; we refer readers to each of the separate articles for elaboration on methodologies and techniques employed in the initiatives discussed.

The final section of the article responds to the third question, reflecting on our experience and offering suggestions about the pursuit of partnerships to build global HRH capacity. The methods of data collection and analysis are described below.

### Data collection and analysis

The data collected for the description of the partnership was derived directly from the experience of each member of the authorship team representing each of the disciplines and constituencies from within the various organizations that participated in the partnership, including managerial staff and students from both the North and the South. All of the researchers were themselves active participants in this collaboration for at least 5 years, and some, for more than 10 years. As noted by others [41, 42], the use of participant-observation has advantages over second-hand accounts and can provide valuable insights through reflection. As we were the ones most directly involved with all the components of the research - from the formation of the collaboration, to planning the research agenda and designing the projects, to collecting and analyzing the data and synthesizing results for decision-makers and scholarly venues – our own perceptions, synthesized through the process of writing this article, provided the main source of data. The description was aided by reference to the over 10 publications already published from our collaboration [20, 43–52].

The context-mechanism-outcome method [53] applied to delineate the mechanisms employed to achieve success is the general approach used in “realist reviews” [53], which, as described by Spiegel et al. [20], is a strategy for synthesizing research that has an explanatory rather than judgmental focus. In realist evaluation, to infer a causal outcome (O) between two events, one needs to understand the underlying mechanism (M) connecting them and the context (C) in which the relationship occurs, with the basic evaluative question of ‘what works?’ replaced by ‘what is it about this program that works for whom in what circumstances?’[54, 55]. As for the first research question, several sources of information were used for constructing the analysis, supplementing participant observation with information available through the myriad of studies we published.

The method of analysis used to address the third question is analytical induction, whereby tentative hypotheses were constantly refined, altered or abandoned in light of the data collected, in this case, the discussion amongst the ten co-authors of this article. Specifically, to identify ‘lessons learned’ we adopted an iterative reflexive approach that reveals the personal perspectives and socio-political contexts that shape our various constructions of meaning [56].

## Results and Discussion

### What model characterizes our partnership?

#### *Contextualizing the origin of the partnership: A Northern collaboration with practical experience*

The SARS experience revealed systemic health sector weaknesses that left health workers, patients and the general public vulnerable. In particular, Canadian occupational health and infection control experts learned that integrated occupational health-infection control training was needed to develop a positive safety culture that served the workforce and patients alike; and that a more integrated surveillance approach was required. This led to a partnership to develop information and communication technology (ICT) tools, including animated training materials and a web-based information system to systematically reinforce surveillance of workplace conditions and workforce health. The research conducted in line with this concern illustrated the need for better workplace inspections, and an integrated workplace audit tool was then developed to supplement worker questionnaires and the ICT innovations. The products developed were heralded as innovative, leading to their adaptation and use internationally [39]. Moreover, the transformations that needed to take place at the levels of the individual health worker, the healthcare facility and the health jurisdiction, were documented and analyzed as a key part of the learning process. This partnering initiative was recognized in 2011 by the Canadian Institutes of Health Research (CIHR) and the Canadian Medical Association Journal (CMAJ) as one of the six top achievements in Canadian health research that have had a significant impact on health, healthcare and health research [40]. The practical experience and insights gained in addressing a serious infectious disease threat gained in this interdisciplinary university researcher-hospital practitioner collaboration in occupational health and infection in the “Global North” laid the basis for work in the Global South. Importantly, as discussed below, the experience gained in the Global South was a key factor in strengthening this collaboration in the North, building on insights gained from South African research colleagues and health practitioners.

#### *Contextualizing why and how the Canadian-South African partnership developed*

In South Africa, HRH is characterised by inequalities between and within provinces, as well as rural and urban locations within the public sectors [57]. In this country, annual per capita expenditure on health ranges from $1,400 USD in the private sector to approximately $140 in the public [6]. The national public health sector, staffed by some 30 % of the country’s doctors, remains the sole provider of health care for more than 40 million people who are uninsured and who constitute approximately 84 % of the national population [58].

In 2010, 49 % of medical practitioner posts and 46.3 % of professional nurse posts were vacant [59], despite the growing dual epidemic of HIV and TB increasing the demand for healthcare [33, 60]. South Africa’s high HIV prevalence [61] has fuelled the epidemic of TB [62]; South Africa’s TB incidence is still among the highest in the world at approximately 860 per 100,000 [63]. Given the HRH shortage in South Africa, together with elevated infection risks faced by health workers, the need to promote a healthier and safer healthcare work environment is particularly critical [44, 64].

When representatives of the South African government learned about the Canadian health sector efforts at a meeting of the WHO collaborating centres in July 2006, they suggested that the Canadian team work with South African institutions to improve occupational health and infection control specifically and the health and safety in the healthcare workplace more generally. The National Institute for Occupational Health (NIOH), a WHO collaborating centre, together with a Department of Health representative, invited the Canadians to Johannesburg in November 2006, and convened a meeting attended by authorities from across the country, including provincial as well as national personnel, and unions.

The consensus of the Canadian-South African team was to pilot a project in one hospital in South Africa, and promote joint learning about the challenges and opportunities for creating desired improvements as well as for testing materials and processes. Pelonomi Hospital in the Free State province was chosen, as it met the criteria of a) having an existing occupational health unit active in infection control and eager to take on a new challenge (including implementing a surveillance system); b) supportive management; c) a functional joint health and safety committee comprising management and worker representatives; d) strong support from the provincial Department of Health’s Provincial Occupational Health Unit; and e) a local university that could serve as a research partner [43, 65]. This hospital became the main site for re-engineering of the Occupational Health and Safety Information System (OHASIS) based on the experience of the Canadian web-based system [20, 45–47].

In light of the previously mentioned personal and resource constraints, what made this undertaking feasible was the commitment of NIOH, with its technical expertise and core capacity to co-develop the needed ICT innovations alongside the Canadian partners. The mandate of NIOH is to provide occupational health and safety technical support across all sectors of the economy to improve and promote worker’s health; to conduct research to further occupational health; and to provide teaching and training in occupational health.

With OHASIS and related training underway, attention shifted to strengthening the skills of front-line workers to prevent their workplace acquired infection with HIV and TB. Feasibility and pilot studies were begun in Free State Province [48], to assess the use of the OHASIS information system for this purpose. NIOH also began to develop a model occupational health program targeting TB infection control at one hospital in Gauteng Province. Furthermore, NIOH quickly seized the opportunity to extend the use of the OHASIS system to the network of 349 laboratories employing 6700 staff at the affiliated National Health Laboratory Service (NHLS) and subsequently began discussions for its further implementation.

Johnson and Wilson [10], examining a partnership between practitioners in the United Kingdom and Uganda, emphasized the mutuality in what they called “North–South/South-North” partnerships. Holmarsdottir (2013) also stresses mutual benefit in North–south-South collaborations in which countries from the South enter into partnership with each other as well as one or more Northern partner. Our case involves the mutuality of North–South/South-North benefit, but the key aspect of the approach we adopted was a Northern partnership working with a strong Southern partner, and together working with less well-resourced Southern partner within the same country. As such, we characterize our model as a North–south-South partnership, albeit only two countries were involved. It is however noteworthy that interest has indeed been expressed by additional partners in other African countries, and this work is now underway in Zimbabwe and Mozambique as well, with NIOH continuing to play a strong role.

### What success was achieved and how? The local projects, their rationale, their mechanisms and their outcome

The initiatives we undertook are discussed briefly below, highlighting the scale of implementation, as summarized in Table 1. It should be noted that this collaboration, from its onset, had a strong research component, in line with the commitments of the Canadian and South African partners who initiated it. From this perspective, the role of research trainees has been central, with a distinct focus on implementation science i.e. what contributes to practices working and why. Important to the success of these initiatives has been the strong role played by students both from Canada and South Africa – with each project tightly linked to student-led initiatives for dissertations or major papers for their respective academic projects. This aspect is highlighted in the descriptions below.

**Table 1:** Projects within this South African-Canadian partnership: Interventions implemented for impact at different scales^a^

Scale project	MICRO outputs- health worker e.g. Professionals, allied workers	MESO outputs- workplace e.g. Hospital, laboratory, clinic	MACRO outputs -jurisdiction e.g. Province, national entity
1. Occupational Health and Safety Information System (OHASIS)	Health workers familiar with procedures to protect their health & safety; health workers better able to promote healthy work environments.	**System established in numerous hospital workplaces, clinics & laboratories to provide information to OH professionals & facility decision-makers to support a healthy work environment.**	Policies & technical support provided to provincial decision-makers for sustainably maintaining healthy work environment; & technology transfer to national partner for ongoing work with provinces.
2. Certificate programme for training health workers (see Table 2 for details)	**Health workers more skilled and confident to prevent & manage HIV and TB risk in the workplace, including how to conduct & evaluate workplace interventions.**	Systems and innovations implemented to better prevent and manage blood-borne and airborne infectious disease (especially TB) in workplaces of the trainees.	Policies, support and oversight for actions to prevent & control TB risk in the workplace conveyed to provincial authorities through presentations by trainees.
3. TB infection control tools, policies and procedures	Health workers more skilled and confident in taking steps to prevent and control TB risk in the workplace, including how to conduct workplace interventions.	**Policies to prevent and control TB workplace risk; & systems implemented (pilot in 28 hospitals in Free State, plus one in Gauteng & one in Western Cape)**	Policies and technical support for actions to prevent & control TB risk in the workplace directly discussed with Provincial executive to be implemented beyond the hospital level, and with direct coordination with national policies. (See Table 3 for Free State policies developed)

^a^The scale where primary emphasis for each project is targeted is noted by bold; i.e. for project #1, while all levels were affected, the primary focus of intervention is at the meso (workplace); project #2 the focus was on training health workers, so the scale is micro (individual) although clearly with the intent of having impact at the workplace and ultimately provincially and nationally; project #3 targeted both the hospital and provincial level in its implementations

- Building infrastructure: The Occupational Health and Safety Information System (OHASIS)
  Despite legislation that establishes processes (e.g. joint management-worker committees) to oversee the creation of safe and healthy working conditions, the information and capacities needed to take on this challenge have remained sorely lacking. To study the usefulness of applying a computerized information system to reinforce health and safety practices, OHASIS modules [66] were refined and co-developed in workshops and then introduced for use in three hospitals in Free State. Workplace assessments (audits) to record deficiencies and model practices, as well as to formulate recommendations for action, were conducted initially by joint North–South teams as a key capacity-strategy, resulting in the preparation of a collaboratively developed paper-based and electronic “Workplace Assessment Field Guide for Health Care”[67] which became the basis for report forms and future training.
  Challenges were of course experienced in pursuing this initiative. For example, limited technical capacities to support computerized systems (including inadequate bandwidth) and restricted staff access to computers were frequent sources of frustration. Nevertheless, most occupational health nurses, infection control personnel, health and safety committee representatives and managers interviewed indicated that the introduction of the “system” served to draw attention to the processes needing attention, with the anticipation that the full value of the integrated system will be realized with time. While local capacities for supporting OHASIS were limited in the Free State hospitals, the roll-out of the system throughout NHLS actively supported by the NIOH, went quite smoothly, progressing from a paper based to an online system with more than 300 employees trained and accessing the system. Recognizing this, an agreement was enacted between the host Canadian institution and NHLS/NIOH for a transfer of the computer code without cost, under a Creative Commons Licensing agreement, ensuring that further development could be led directly by South Africans. Shortly thereafter, NIOH prepared a lighter and updated version with the assistance of the original developers.
  At NHLS, the information system was found to be effective and efficient in capturing and using information on worker health [68] and the related investigation and reporting of health and safety incidents, as substantiated by surveys of randomly selected employees in 2012 and 2015, revealing increases in feeling “always comfortable reporting health and safety problems to their manager” (up 17 %) and “encouraged to report injuries and illness in the workplace” (up 16 %). This observed efficacy provided impetus for NIOH/NHLS to develop a new module for waste management with new international (WHO) funding that it was able to attract.  Agreements are now being finalized to introduce the integrated OHASIS system across Gauteng province, led by NIOH which has taken over updating and improving OHASIS by creating a streamlined version that is easier to run in the South African context (responding to technical challenges identified in Free State) as well as training materials to support implementation scale-up.^1^ The system is also about to be introduced in one hospital in Western Cape as well and discussions are underway for further roll-out in Free State. To refine the implementation of the system, two South African graduate students are playing a major role. A Master’s of Business Administration student who is directly involved in the project roll-out in Gauteng is conducting a study on how to design reporting to meet the needs of managers and a Medical Resident in Occupational Medicine is focusing on how to implement a surveillance system to decrease occupational TB in a hospital in Western Cape; while a Canadian doctoral student is also involved in studying the factors determining successful implementation in each of the settings across South Africa. Notably, co-development of OHASIS, involving Canadian and South African developers is continuing, with the explicit agreement that modules developed or improved upon will be shared freely with all parties internationally.
- Multi-directional learning and the training of healthcare workers to implement occupational health and infection control
  To improve local capacity to conduct and evaluate workplace-based HIV and TB prevention interventions, and to empower healthcare workers to serve as “agents of change” within high-risk workplaces, a 1-year certificate program was collaboratively developed by the Canadian infection control and occupational health partners together with colleagues from NIOH and personnel from the Free State Department of Health and the University of Free State. Thirty-one participants - mostly occupational health nurses, infection control practitioners, and health managers - formed eight groups with the task of designing and conducting projects for improving occupational health and infection control in their workplaces. Each group was assigned Canadian and South African mentors. Table 2 outlines the various projects that were undertaken. Many of the program’s “graduates” continued to play an active role in the larger research program that subsequently developed [47]. A South African graduate student assisted in implementation in Free State; several Canadian graduate students worked with each of the various projects [48–51] helping to implement this program, with one Canadian graduate student writing his Master’s thesis about this program overall [48]. One of the South African trainee projects (Table 2 row 6), led by a nurse from a rural hospital in Free State, was subsequently published in a peer-reviewed journal [50].

**Table 2:** Synthesis of projects implemented through the training programme in Free State, South Africa ^a^

Project title	Trainees	Setting	Objective	Methods	Key findings
Investigating TB infection control practices in Outpatient Department (OPD) (http://med-fom-ghrp-spph.sites.olt.ubc.ca/files/2012/09/FINAL_group-5-powerpoint-presentation_May15.pdf)	SE Mmutle, MR Morake, ME Moea, NF Jacobs	OPD at Pelonomi Hospital (large regional referral centre, Bloemfontein)	- To make recommendations to management regarding TB infection control	- Self-administered questionnaires assessing TB infection control knowledg & practice - Workplace assessment checklist to assess hazards and best practice	- Only 24 % of HCWs reported that they are screened annually for TB - 47 % answered questions related to personal protective equipment correctly - 84 % reported asking coughing patients to practice respiratory etiquette - Sputum collection area was inaccessible
Reducing the risk of DOTS supporters acquiring TB during home visits (http://med-fom-ghrp-spph.sites.olt.ubc.ca/files/2012/09/Group-2-final-presentation.pdf)	N Nyembe, N Jacobs, D Mofokeng	Neighbourhood communities (Bloemfontein & Welkom)	- To identify and assess strategies used by DOTS supporters from NGOs to reduce TB transmission - To assess the impact of a TB infection control training intervention	- Pre & post questionnaires assessing TB knowledge, attitudes & beliefs - Interviews assessing TB knowledge & practice - 2-day training programme - Infection control practices audit	- Overall improvement in levels of knowledge, attitudes beliefs regarding TB - Lack of administrative controls & use of personal protective equipment identified - No N95 respirators available - Health & safety problems were reported to coordinators but not followed up
Creating a safe environment for patients and staff in the bronchoscopy theatre (http://www.ghrp.ubc.ca/files/2012/09/Group-4-presentation-function-17h-may-16th.pdf)	HM Madiehe, ML Magerman, MJ Morweng, ME Motloheloa, A Smuts, TC Walaza, EA Wiese	Bronchoscopy theatre at Universitas Academic Hospital (large tertiary hospital, Bloemfontein)	- To assess compliance with TB infection control guidelines - To make recommendations to minimize TB exposures for staff and patients	- Structured observations to evaluate infection control practices in the bronchoscopy theatre & waiting room - Checklist-based environmental risk assessment of the theatre	- Improvement rate in infection control compliance from 46 % to 83 % - Environmental risks identified included overcrowding, poor ventilation, lack of hand-washing supplies & cluttered surfaces
Strengthening the OHC for the management of TB in the health care workplace (http://med-fom-ghrp-spph.sites.olt.ubc.ca/files/2012/09/Group-7_May16_5-30pm.pdf)	L Benson, DA Kololo, NJ Sidyiyo, MW Moliko, J Nkhatho, NW Phandle, H Langfoot	Universitas Academic Hospital	- To strengthen the workplace TB programme in the OHC	- A feasibility study was conducted to inform development of a cough registry - Occupational health & safety tools were developed: the cough registry, permission slips, a plan for diagnosis and treatment of HCWs with TB - Operational managers were trained on the use of the cough registry	- These activities led to an increase in utilisation of the Occupational Health Clinic - Confidentiality was identified as a barrier
Improving infection control and safety practices in the Central Laundry: A baseline assessment (http://med-fom-ghrp-spph.sites.olt.ubc.ca/files/2012/09/Group-8-presentation_-16-May.pdf)	MM Litsoane, KD Moeketsi	Free State Provincial Laundry Facility (Bloemfontein)	- To assess occupational health & infection control knowledge & practice - To minimize workplace exposures/hazards	- Self-administered questionnaire assessing occupational health & infection control knowledge & practice	- Hepatitis B vaccination reported by 85 % - 90 % reported no training was received on needle-stick injury prevention - 72 % reported never using eye protection - 82 % knew how to contact the health & safety representative, but only 56 % reported doing so
Reducing blood and body fluid exposure in the workplace (http://med-fom-ghrp-spph.sites.olt.ubc.ca/files/2012/09/revised-Litsitso_Nkoko.2012.Graduation_presentation.pdf)	L Nkoko	Thebe District Hospital in Thabo Mofutsanyana (mid-sized rural hospital)	- To determine knowledge, attitudes & practices of HCWs regarding exposure to blood and body fluids	- A questionnaire investigating BBF exposures, reporting of exposures, & HCWs’ knowledge of infection control and occupational health resources was distributed to all HCWs in 11 high-risk departments in the hospital	- Many respondents did not know enough about BBF exposures actions. - HCWs take immunisation for Hepatitis B seriously - Most take precautions to avoid BBF exposures; most report exposures. - OHS representatives need to be more proactive within their units. - More training is needed on sharps disposal & the importance of using personal protective equipment.
Improving utilisation of workplace HIV and AIDS programme for healthcare workers at Pelonomi Hospital (http://www.ghrp.ubc.ca/files/2012/09/Group-1-presentation-for-graduation-working-copy-11.FINAL_.pdf)	N Brandsel, M Ntlola, N Myeko,F Tlhapuletsa	Pelonomi Hospital (large tertiary hospital in Bloemfontein)	- To understand why the Occupational Health Service (OHS) is under-utilised for the HIV & AIDS program in order to determine what can be done to improve the service.	- Self-administered questionnaires consisting of both closed & open-ended questions. - The questionnaire was piloted, translated & back translated before distribution to a stratified sample of HCWs representing all categories of occupation, sex, race & age	- 57.6 % knew that HIV treatment is available at the OHS - 71.3 % agreed that occupational health (OH) practitioners are well trained to offer HCT, while 70.4 % agreed that OH practitioners encourage people to use the ORS unit for HIV and AIDS services - 71.2 % believed that confidentiality is maintained at the OHS unit most or all of the time, however, when asked what factors explain why HCWs do not access HIV services at OHS unit, most stated that they feared that confidentiality will not be maintained (37.3 %)

^a^ For more information on the Certificate Programme offered at the University of Free State, through the assistance of the partnership details see Liautaud A, Yassi A, Engelbrecht M, O’Hara L, Rau A, Bryce E, Spiegel J, Uebel K, Zungu M, Roscoe D, et al.: Building Capacity to Design, Implement and Evaluate Action Research Projects to Decrease the Burden of HIV and TB in the Healthcare Workforce: A South African- Canadian Collaboration. Open Medicine 2013, 7:s33 [48]. For Abstracts and presentations for each project see the weblinks noted. For Project #6, see L. Nkoko et al. 2014 [50]
  Stigma and concerns about confidentiality are often cited as barriers to uptake of HIV counselling and testing (HCT) by health workers, but without much data to reveal underlying reasons [69]. This lack of empirical evidence hinders efforts to improve utilisation of such services. Spurred by the South African trainees’ finding that between January and May 2011 only 121 of its more than 1900 health workers had accessed the HCT service, a trainee group project (see Table 2 row 7) addressed this issue, as did a Canadian Masters student’s thesis [49]. More than one-third of health workers surveyed (38.5 %) indicated they believed that there was HIV stigma in the workplace. Additionally, nearly 40 % of all survey participants indicated they would not use the occupational health unit at their workplace due to fear that confidentiality might not be maintained [49].
  Building on the work of the South African trainees, another Canadian student – working on his Master’s in Health Administration - conducted a best–worst scaling choice experiment to quantify attributes that may influence a health worker’s choices as to whether and where to be screened for TB. This study included 2 focus groups, key informant interviews, and distribution of a questionnaire at two hospitals in Free State. Analysis of results found that to improve uptake of TB screening by health workers, programs should be free, guaranteed, confidential, with minimal waiting times, and available at the workplace [52].
  With documentation that stigma is a major issue, yet another Canadian Masters student worked with local staff to inform the development of a stigma reduction intervention as part of a large multicomponent trial being planned. Relevant results of four feasibility studies conducted [47] were analyzed along with the literature. The findings stressed that a stigma reduction campaign must address community and structural level drivers of stigma, in addition to individual level concerns [46].
  The North–South partnership at the Gauteng Hospital (described below) is giving rise to at least two further academic projects at the Doctoral and Masters levels – for South African students. The process of involving Canadian graduate students as well as South African graduate students to work with healthcare worker trainees in LMICs facilitated multi-directional learning – and is an aspect of North–South collaborations that we believe is extremely important to explicitly recognize as a mutual benefit.
- Implementing occupational health infection control programs in a high TB-burden hospital in Gauteng and in Free State
  NIOH took on responsibility for implementing a workplace health programme to protect heath workers at a hospital in Gauteng province, with the North–South partnership collaboratively designing and implementing a TB infection control programme (ICP) in this hospital. The TB ICP has three major components: 1) training of hospital personnel and implementation of a TB workplace assessment (audit) to identify hazards for airborne diseases, as well as identify best practice where applicable (recommendations are being implemented and will be reassessed); 2) a survey of healthcare workers’ experience and perceptions about TB infection control at the hospital which revealed high TB exposure risk, lack of TB infection control training and a threefold greater risk of TB compared to the general population [45]); and 3) methodology for quantifying TB bacilli in the air [51] with the findings then used to sensitise management to the need to implement control measures.
  The partnership succeeded in elevating attention to TB infection control and occupational health and safety overall, with occupational health and safety now reporting directly to the hospital CEO. As well, the partnership has increased the knowledge and confidence among infection control and occupational health workers, highlighted the plight of healthcare workers at the hospital, and assisted healthcare workers to advocate for their right to a healthy and safe working environment.
  While the Gauteng project was underway, new integrated policies and standard operating procedures were also developed in Free State Province, with the North–South partnership engaged in implementing and evaluating impact at 28 hospitals across the province [47]. Table 3 summarizes the new policies that were developed and implemented, with comments regarding challenges encountered and accomplishments. Importantly, while progress was made, inadequate staffing remains a challenge that will require ongoing attention. We are also undertaking further work to address stigma [70], experimenting with new participatory including arts-based methods [46, 71].

**Table 3:** Impact of the partnership on policies and practices in Free State

Before the partnership	Since the partnership became actively involved in the Free State Province
1. Management involvement was limited, and not in compliance with legislation^a^	• CEOs of hospitals recognized their legal obligation and new policies were approved by the Free State Head of Department and Member of the Executive Council for Health in 2013, starting with the establishment of health & safety representatives and committees
2. Policies were not based on evidence.	• New policy on management of TB at the workplace developed
	• New policy on workplace assessment developed
3. Inadequate staff resources were allocated to this area	• Four new Occupational Health Nurse Practitioners (OHNPs) were appointed to provide improved health services for the workforces.
4. Programme coordination was a gap, with limited working together of different professionals	• The Partnership established programme coordination and working together of different professionals (Infection Control [IC] practitioners, TB Coordinators and OHNPs),
	• There are regular meetings at Provincial level of these different professional groups who are now working together
5. TB and HIV management at OHC was not well utilised by healthcare workers; OH nurses were not trained or authorized to prescribe TB and HIV treatment nor other PHC treatment	• All OHNPs are now authorised to prescribe TB and HIV treatment as well as other Primary Health Care (PHC) treatment and medication issues by hospital Pharmacy
	• Improved healthcare workers usage of TB and HIV management at OH clinics (OHCs) - now free treatment available
	• Health workers can get medication at own GP if preferred, come for follow-up and get service free at OHCs.
	• Perception among OH staff that there has been decreased disability leave and staff leaving due to disability, and fewer employees suffering work related diseases and injuries (although this is in the process of being ascertained more rigorously)
6. No reliable electronic database for capturing information; no standardised medical surveillance tool; and no standardized approach to identifying and recording workplace hazards	• OHASIS brought easy-to-use system, which specified data to be collected to inform Management of need for future policy reviews and/or implementation measures to better protect health workers.
	• Training on OHASIS for OH/IC professionals as well as health and safety representatives, using a structured approach to code risks/hazards, made it easy to understand types of hazards
7. Very limited research capacity for occupational health and infection control intervention studies.	• Research capability improved through 1-year Certificate course for OH and IC personnel
	• Research output of short course gave evidence base of workplace conditions at different facilities
	• Workplace conditions were perceived to have been improved through specific targeted efforts and reports to Managers and CEOs resulting in approvals for further research.

^a^The Occupational Health and Safety Act, 1993, states that occupational health and safety is the legislated responsibility of every employer including the public hospitals and clinics (OH&S Act, 1993). A National Health Plan for South Africa was prepared by the African National Congress (ANC) with the technical support of World Health Organization and (United Nations Children’s Fund) in May 1994. The ANC initiated a process of developing an overall National Health Plan based on the Primary Health Care Approach; occupational health and safety (OHS) was included in the Plan. Specifically, Chapter 14 of the *White Paper for the Transformation Of The Health System In South Africa* (1997) was entirely devoted to Occupational Health; this document later became the National Health Act no. 61 of 2003, with Chapter 4 section 25 (2)(r) stipulating that the Head of Health in the province must provide occupational health. The key strategy for delivering OHS services for the Department of Health is through Occupational Health Units attached to health facilities. It was also indicated that Provincial OHUs should be established as part of provincial health services to coordinate and monitor OHS, and to oversee training, information, surveillance, assessment of compensation for occupational disease and injury, advice on workers’ rights to compensation, research, and specialised medical services

### What are the lessons about determinants of success in partnerships?

Our partnership aimed to strengthen capacities for improving working environments of health workers, with emphasis on reducing workplace TB transmission as a high priority. As was shown in Table 1, at the micro level, our projects targeted personal knowledge and skills of health workers to not only follow proper procedures to better protect themselves and fellow workers from harm – but to develop skills for advocating for change at the meso (workplace infrastructure) and macro (government policy and resources) levels. At the workplace level, we attempted to strengthen information systems for providing active surveillance, reporting and prevention. At the macro level, we focused attention on the establishment of policies and procedures to enable effective and sustainable provision of healthier workplaces and successfully advocated for increased resources to be allotted to meet identified needs.

Valuing experiential understanding as well as scientific-technical expertise, we relied on fostering peer-to-peer interactions in complex interdisciplinary and inter-professional processes in both the Northern and Southern settings. The priority that was assigned to research throughout the exercise facilitated attention to developing and disseminating innovations. We also explicitly acknowledged that partners had differing albeit complementary expectations. Table 4 summarizes these differences with respect to research and producing new insights; teaching and learning; and service and practice.

**Table 4:** Different expectations in North- South-South collaboration for building HRH

Expected outputs activity area	Expected outputs for Northern partner	Expected outputs for leading Southern partner institution	Expected outputs for local Southern health system/hospital partners
Research & New Insights	∎ Scholarly publications (peer reviewed)- with lead authorship on some and co-authorship on others ∎ Further grant funding	∎ Scholarly publications (peer reviewed)- with lead authorship on some and co-authorship on others ∎ Manuals and working papers for local implementation, promoting problem-solving and surveillance, with potential for scale-up	∎ Participation in scholarly publications usually as co-authors ∎ Manuals and working papers for local implementation, promoting problem-solving and surveillance ∎ Receipt of simple knowledge-translation products that can be used for local impact
Teaching & Learning	∎ Training of graduate and post graduate students leading to successful project papers/theses of Northern students ∎ Professional mentoring of Southern leaders ∎ Respectful bi-directional capacity-building	∎ Training of graduate and postgraduate students leading to successful project papers/theses of Southern students ∎ Development of local health professionals and paraprofessionals in train-the-trainer approach ∎ Respectful bi-directional capacity-building	∎ Training of healthcare workers leading to greater confidence in fulfilling their healthcare responsibilities ∎ Development of local health professionals and paraprofessionals and trained trainers who can continue the work ∎ Respectful bi-directional capacity-building
Service & Practice	∎ University/hospital service (curriculum development, sharing lessons) ∎ Participation in professional associations - national (Northern) and international ∎ Insights from applying policies and procedures in high risk circumstances	∎ Fulfilling institutional mandate ∎ Participation on national committees for policy development and implementation ∎ Professional associations - national (Southern), international ∎ Surveillance of overall effectiveness of policies and procedures	∎ Fulfilling hospital/health system mandate, with improved policies and practices implemented in the workplace ∎ Participation on local committees for policy development and implementation ∎ Professional associations - local ∎ Documentation on effectiveness of policies and procedures

In recognizing the complexities in successfully implementing interventions at different scales, our attention was drawn to different types of partnership. As shown in Table 5, a traditional model for North–South partnerships is characterized by Northern experts working with local Southern practitioners in resource-limited settings. This model allows for direct assistance but is limited in sustainability; also Northern experts may not have sufficiently in-depth understanding of the local reality, and may be more focused on addressing scientific-technical dimensions than practical implementation process challenges- particularly those at the macro level. Here cautions about hegemonic knowledge production [12] are particularly warranted.

**Table 5:** Characteristics, strengths and challenges of different partnership models

Type of partnership	Characteristics	Strengths	Challenges
Model 1: Northern experts – Local Southern partners (North–South)	Northern experts work directly with local health practitioners in resource-constraint settings	Potential for knowledge from the North to be made directly available to practitioners on the frontlines;	Practical contextual understanding of the Southern reality may be limited and sustainability uncertain
Model 2: Northern experts working with strong lead institution based in the South that has a mandate to work to build capacity in its jurisdiction (North–South)	Northern experts work directly with counterparts at the national level or in lead Southern institutions, who, in turn, work with local health practitioners in local resource-constraint settings	Sustainability enhanced with leadership reinforced in South jurisdiction; capacities for technology transfer enhanced	Mutuality limited by unclear grounding in practical realities of Northern partner; with limited ability for mutuality at practitioner level; limits to bi-directional learning
Model 3: North–South-South Community of practice	Practitioners and researchers from the North and South work together with local practitioners	Ability to develop, share and analyse implementation at different scales; enhanced bi-directional (or actually tri-directional) learning	Complexities in sustaining tripartite relationship.

A second model links Northern experts with a strong Southern partner that, in turn, works with local Southern less well-resourced communities. This is a more sustainable option than the first, as the capacity-building with the strong Southern partner is more likely to result in the ability to implement appropriate and feasible policies and systems. However, the lack of direct involvement of the Northern experts with the local Southern communities limits the extent to which bi-directional learning can occur, to strengthen the overall effort. Here cautions about maintaining mutuality [10] need to be recalled.

The model we adopted can be characterized as a community of practice in which the members of the Northern partnership work directly not only with the stronger Southern partner but also with their local counterparts. In this model, the value of peer- to-peer interactions can be fostered not only at the micro and meso levels but also at the macro scale - essential for effectively addressing implementation challenges. The consciousness-raising that occurred through this partnership has already led to calls to improve the way global health is taught in medical schools in the North [72].

Johnson (2007)’s conceptualization of communities of practices as “an action learning space [with] mutual engagement, shared enterprise and shared repertoire as dynamic processes subject to a range of social relations and differences between actors” provides a useful perspective for considering how the partnership principle of “joint learning” that is put forward by the Tropic Health and Education Trust (THET) can be operationalized, especially recognizing the different scales that must be addressed for sustainability (another THET principle) [11].

In considering the mechanisms and outcome achieved in different contexts in which we worked, we identified five key features of the partnership that we see as having been essential in what we accomplished:

First, the Northern *HIC partners’ practical experience in addressing similar issues* was invaluable in establishing a peer-to-peer partnership in which both HIC and LMIC colleagues had “real world” experience. In our case, the Canadians developed considerable collaborative experience in addressing SARS, then later H1N1, and created a range of training materials, information systems and integrated policies. This was not only important in building the expertise of the Northern partners to apply in the collaboration with Southern partners, but highlighted the relevance of the Southern-based experience to also addressing problems in HICs.

Secondly, the active leadership of *a strong national Southern partner* with experience in training, surveillance and policy development for the healthcare sector, and a mandate to work with local partners for implementation, was crucial. In our case, NIOH and the NHLS fit this criterion perfectly. This enabled not only North–South collaboration, but sustained South-South collaboration, in that the “national” Southern partner has been able to continue the work with “local” Southern healthcare partners. While there is a growing literature on North–South-South collaborations, this generally relates to a strong Southern country working with a weaker Southern country; our experience illustrates that the same principles apply within a single Southern partner country.

Thirdly, *the mutual nature of the learning* was an explicit goal of the activities undertaken – a feature we think has been important in our success. Northerners always learn and gain from such experiences as much as they teach and offer the Southern partner. In our case, the extensive involvement of both Canadian and South African graduate students working with South African healthcare trainees played a large role in all activities undertaken. The interdisciplinary community-university partnerships in both the HIC and LMIC combined research, service and training; *mutual appreciation of the varied needs* was an essential part of the knowledge exchange.

Fourthly, our partnership recognized that it was not sufficient to provide training and other measures to strengthen individual knowledge and skills, but that we also had to address organizational infrastructural needs and governmental policies. As such, the *recognition of the need for interventions at micro, meso, and macro level was of critical importance*. Indeed, we believe that emphasizing the social determination of health [38] at all levels is paramount.

Finally, the model adopted - of establishing a community of practice, involving HIC researchers and practitioners along with LMIC researchers and practitioners working at different scales (training individuals, strengthening workplace infrastructure, achieving improved policies and resource allocation from government) - synthesizes the lessons learned. The key message, therefore, is that investing in developing a multi-scalar *community of practice*, centered on strengthening a key LMIC institution that can sustainably work with local partners, is thus especially useful; we believe this model and these key features can be applied not only to improving occupational health and infection control, but sustainable health systems strengthening more generally.

## Conclusions

Working conditions for health workers worldwide have been undergoing rapid change, with new methods for diagnosis and treatment of diseases, combined with rapid communication technology, improving global ability to disseminate new knowledge remarkably effectively. On the other hand, economic globalization is severely straining healthcare resources, preferentially benefiting richer countries [73, 74]. Health worker migration [74], trends to deregulation [73, 75] and weak health systems also impact human resources for health, with the recent Ebola outbreak representing only the tip of the iceberg. Thorsteinsdottir and colleagues [13], drawing on their own experience, emphasize the urgent need for international collaboration to address the ever-faster spread of infectious diseases and outline some of the challenges encountered, including lack of research resources in Southern public sector institutions and technological challenges. The five key aspects we identified as crucial to success add to some of the proposed actions they articulate, with our experience highlighting the usefulness of a bi-national North–South-South model. We believe that this approach can have widespread applicability, providing mutual benefit to all parties involved.

## Footnotes

1 Training material and an introduction to the OHASIS system are available at http://ghrp.ubc.ca/products/ and will be available on an NIOH site.
